# PCSK9 Inhibition: Insights From Clinical Trials and Future Prospects

**DOI:** 10.3389/fphys.2020.595819

**Published:** 2020-11-16

**Authors:** Julius L. Katzmann, Ioanna Gouni-Berthold, Ulrich Laufs

**Affiliations:** ^1^Department of Cardiology, University Hospital Leipzig, Leipzig, Germany; ^2^Polyclinic for Endocrinology, Diabetes, and Preventive Medicine, University of Cologne, Cologne, Germany

**Keywords:** PCSK9, LDL cholesterol, atherosclerosis, coronary artery disease, evolocumab, alirocumab, small interfering RNA, inclisiran

## Abstract

In 2003, clinical observations led to the discovery of the involvement of proprotein convertase subtilisin/kexin type 9 (PCSK9) in lipid metabolism. Functional studies demonstrated that PCSK9 binds to the low-density lipoprotein (LDL) receptor directing it to its lysosomal degradation. Therefore, carriers of gain-of-function mutations in *PCSK9* exhibit decreased expression of LDL receptors on the hepatocyte surface and have higher LDL cholesterol (LDL-C) levels. On the contrary, loss-of-function mutations in *PCSK9* are associated with low LDL-C concentrations and significantly reduced lifetime risk of cardiovascular disease. These insights motivated the search for strategies to pharmacologically inhibit PCSK9. In an exemplary rapid development, fully human monoclonal antibodies against PCSK9 were developed and found to effectively reduce LDL-C. Administered subcutaneously every 2–4 weeks, the PCSK9 antibodies evolocumab and alirocumab reduce LDL-C by up to 60% in a broad range of populations either as monotherapy or in addition to statins. Two large cardiovascular outcome trials involving a total of ∼46,000 cardiovascular high-risk patients on guideline-recommended lipid-lowering therapy showed that treatment with evolocumab and alirocumab led to a relative reduction of cardiovascular risk by 15% after 2.2 and 2.8 years of treatment, respectively. These findings expanded the armamentarium of pharmacological approaches to address residual cardiovascular risk associated with LDL-C. Furthermore, the unprecedented low LDL-C concentrations achieved (e.g., 30 mg/dL in the FOURIER study) suggest that the relationship between LDL-C and cardiovascular risk is without a lower threshold, and without associated adverse events during the timeframe of the studies. The side effect profile of PCSK9 antibodies is favorable with few patients exhibiting injection-site reactions. Currently, the access to PCSK9 antibodies is limited by high treatment costs. The development of novel approaches to inhibit PCSK9 such as the use of small interfering RNA to inhibit PCSK9 synthesis seems promising and may soon become available.

## Introduction

Despite the large evidence base for statins, currently available oral lipid-lowering therapies to reduce LDL cholesterol (LDL-C) concentrations are limited, e.g., by muscular symptoms with high statin doses or insufficient efficacy in very high baseline LDL-C levels in patients with familial hypercholesterolemia (FH). Even after the maximally achievable LDL-C reductions of ∼60% with oral lipid-lowering therapies, patients with atherosclerotic cardiovascular disease (ASCVD) are still facing a considerable residual cardiovascular risk attributable to LDL-C (Sabatine et al., 2017a; Schwartz et al., 2018). Therefore, a clinical need exists for pharmacologic options to lower LDL-C beyond what is achievable by current oral medications. The use of genetic information to identify novel treatment targets has always been an attractive idea, but has rarely been so efficiently realized as in the case of the development of monoclonal antibodies (hereafter referred to as “antibodies”) against proprotein convertase subtilisin/kexin type 9 (PCSK9).

The role of PCKS9 in lipid metabolism was discovered in 2003. Carriers of loss-of-function mutations in the *PCSK9* gene were found to have 28% lower LDL-C, accompanied by 88% reduced risk of coronary artery disease (CAD), making PCSK9 a promising treatment target for ASCVD prevention (Cohen et al., 2006). Based on this clinical observation, the concept of therapeutic interventions targeting PCSK9 was rapidly translated into practice. In 2017 and 2018, two landmark trials on the therapeutic inhibition of PCSK9 with antibodies were published: Only 15 years after the discovery of PCSK9, its therapeutic inhibition was shown to reduce cardiovascular risk on top of therapies established over decades, such as the statins (Sabatine et al., 2017a; Schwartz et al., 2018).

The principle of PCSK9 inhibition not only broadened the therapeutic armamentarium available to treat patients with a high burden of residual cardiovascular risk by lowering LDL-C to remarkably low concentrations (e.g., 30 mg/dL in the FOURIER study (Sabatine et al., 2017a)), but also provided insights about the importance of circulating LDL-C levels.

In the present review, evidence from clinical trials with antibodies against PCSK9 is reviewed, and an outlook for novel approaches to inhibit PCSK9, especially with mRNA-targeting agents, is provided.

## Physiology of Proprotein Convertase Subtilisin/Kexin Type 9 (PCSK9)

### PCSK9 and the Regulation of LDL-C Metabolism

The first reports on PCSK9, initially called neural apoptosis-regulated convertase type 1 (*NARC1*), were published in 2003 (Seidah et al., 2003). Soon after, PCSK9 was identified as being involved in lipid metabolism (Abifadel et al., 2003). This observation was based on genetic analyses. Until then, two genetic loci were known to be causal for autosomal dominant FH: the genes encoding the LDL receptor (*LDLR*) and apolipoprotein B (*APOB*). In members of a French family with phenotypic FH in whom mutations in *LDLR* and *APOB* had been excluded, gain-of-function mutations in *PCSK9* were identified as cause for the elevation of LDL-C (Abifadel et al., 2003).

Following this observation, in-vitro studies demonstrated that gain-of-function mutations in *PCSK9* lead to decreased expression of LDL receptors on the cell surface and decreased LDL internalization (Cameron et al., 2006). On the contrary, in some individuals with low LDL-C, loss-of-function mutations in *PCSK9* were identified. These were linked to lower serum LDL-C concentrations and lower risk of CAD (Cohen et al., 2006). Together with the finding that statin therapy induces PCSK9 expression, which could potentially limit the ability of statins to reduce LDL-C to very low levels (Dubuc et al., 2004), great interest in PCSK9 as potential treatment target in ASCVD prevention and treatment aroused.

**FIGURE 1:**
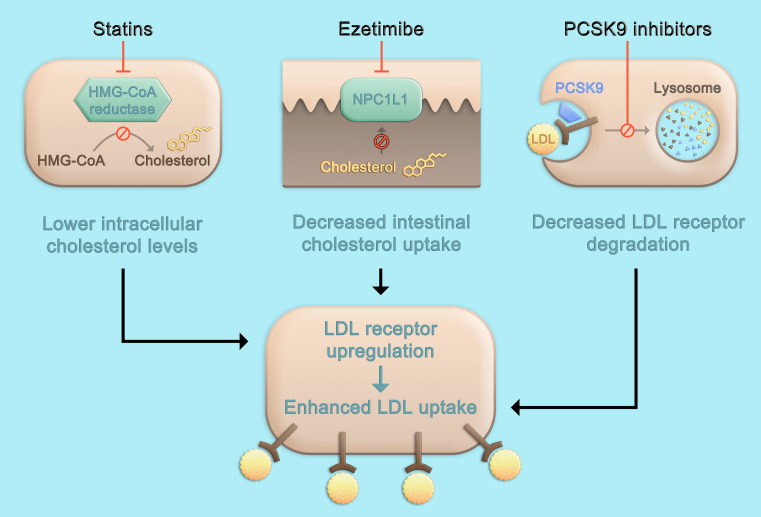
Pharmacologic approaches to lower LDL cholesterol. Statins inhibit the rate-limiting enzyme of cholesterol biosynthesis, HMG-CoA reductase, leading to decreased hepatic cholesterol production. Ezetimibe is an inhibitor of NPC1L1 which facilitates absorption of intestinal cholesterol and therefore selectively decreases dietary cholesterol uptake and hepatic cholesterol supply. The inhibition of cholesterol synthesis or intestinal absorption both lead to an upregulation of the LDL receptor and subsequently, enhance LDL uptake and lower LDL cholesterol serum concentrations. Therapeutic inhibition of PCSK9 also leads to a higher density of LDL receptors on the hepatocyte surface, but not primarily through targeting cholesterol metabolism, but by affecting LDL receptor degradation and recycling pathways (Figure 2). HMG-CoA, 3-hydroxy-3-methylglutaryl-coenzyme A; LDL, low-density lipoprotein; PCSK9, proprotein convertase subtilisin/kexin type 9; NPC1L1, Niemann-Pick C1-like protein 1.

**FIGURE 2:**
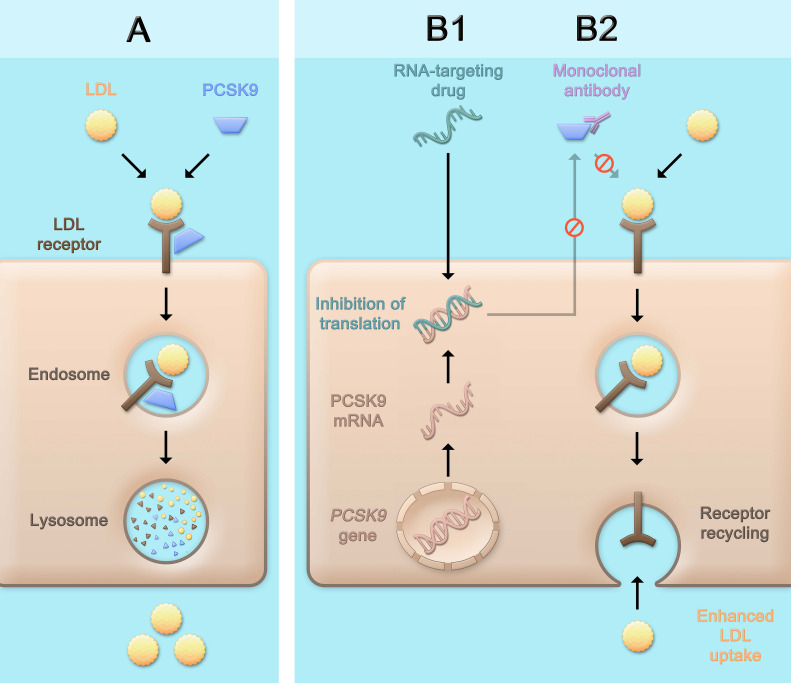
Physiological role of PCSK9 and consequences of therapeutic PCSK9 inhibition. PCSK9 binds to the LDL receptor. After internalization of the LDL receptor bound to PCSK9 (and an LDL particle), the LDL receptor is degraded **(A)**. PCSK9 can be inhibited pharmacologically by using monoclonal antibodies **(B2)** that bind and neutralize PCSK9, or by RNA-targeting drugs **(B1)** which contain an RNA strand complementary to PCSK9 mRNA and lead to the assembly of an RNA-induced silencing complex (RISC) which degrades PCSK9 mRNA for a prolonged period of time and thereby inhibits the production of PCSK9. The consequence of both approaches is a lower concentration of PCSK9 that can bind the LDL receptor, which after internalization more often cycles back to the cell surface and can take up further LDL particles, leading to lower LDL cholesterol serum concentrations. LDL, low-density lipoprotein; PCSK9, proprotein convertase subtilisin/kexin type 9.

While the three classes of LDL-C-lowering medications that beneficially affect cardiovascular risk, i.e., statins, ezetimibe, and PCSK9 inhibitors, lead to a higher density of LDL receptors on the hepatocyte surface and subsequently, enhance LDL uptake and lower LDL-C serum concentrations, the mechanisms by which this is achieved differ (Figure 1). Statins are inhibitors of the rate-limiting enzyme of cholesterol biosynthesis, 3-hydroxy-3-methylglutaryl-coenzyme A (HMG-CoA) reductase, and decrease hepatic cholesterol production. Ezetimibe is an inhibitor of the Niemann-Pick C1-like protein 1 (NPC1L1) which facilitates intestinal absorption of cholesterol and therefore selectively decreases dietary cholesterol uptake and hepatic cholesterol supply (Mach et al., 2020). In contrast, the mechanism by which PCSK9 is involved in LDL-C homeostasis does not primarily involve cholesterol synthesis or absorption: PCSK9 binds to the LDL receptor, which also binds LDL particles and leads to their internalization mainly into hepatocytes. If the LDL-LDL receptor-PCSK9 complex is internalized, lysosomal degradation follows (Figure 2A; Lagace et al., 2006). In contrast, without PCSK9 bound, the LDL receptor can be recycled back to the hepatocyte surface and take up more LDL particles, leading to lower LDL-C levels. This situation occurs more frequently if PCSK9 is lower due for example to therapeutic interventions or if its concentration is lower because of *PCSK9* loss-of-function mutations. Of note, PCSK9 may also have intracellular functions (Glerup et al., 2017), and the consequences of lowering extracellular PCSK9 may differ depending on the way PCSK9 is lowered. In the case of lower extracellular PCSK9, fewer LDL receptors are internalized bound to PCSK9, a smaller proportion of the LDL receptors is degraded, and a larger proportion can cycle back to the cell surface (Figure 2B).

### Consequences of Therapeutic Inhibition of PCSK9

The first successful strategy to inhibit PCSK9 therapeutically is the use of antibodies. The first reports on the use of such antibodies in monkeys were published in 2009 and showed that LDL-C was lowered by 80% after a single injection (Chan et al., 2009). Soon after, antibodies were tested in humans. After successful phase 1 and 2 studies, large phase 3 studies were launched in 2012/2013 (Seidah et al., 2014). Their results are described in detail below.

Anticipating the results of these studies, especially the impact of pharmacologic inhibition of PCSK9 on the risk of ASCVD, a Mendelian randomization study was conducted. Polymorphisms in *PCSK9* were used as proxies to mimic the effects of pharmacologic inhibition of PCSK9, and compared to the effects of genetic polymorphisms in *HMGCR*, the gene encoding the target of statins and therefore, mimicking statin therapy. The effects of the polymorphisms in both genes on ASCVD risk were similar per unit change in LDL-C, and were independent and additive. Furthermore, the risk of diabetes, known to be slightly increased under statin therapy in individuals with prediabetes, was increased with a similar magnitude per unit LDL-C reduction for both the *PCSK9* and *HMGCR* polymorphisms. The results of this study suggested that therapeutic PCSK9 inhibition should lower cardiovascular risk by the same magnitude per change in LDL-C as statin therapy and that the effects may be independent and additive (Ference et al., 2016).

Importantly, however, the metabolic changes induced by treatment with antibodies against PCSK9 and statin therapy are not identical. While both approaches lower LDL-C, triglycerides, apolipoprotein B, and increase HDL cholesterol, statins additionally reduce C-reactive protein (CRP) (Sabatine, 2019), which PSCK9 antibodies do not – although a mouse model suggested anti-inflammatory effects to be involved in the anti-atherosclerotic effects of PCSK9 antibodies (Schuster et al., 2019), and PCSK9 antibodies decrease lipoprotein(a) by mechanisms that are not fully understood, whereas statins do not change or may even slightly increase lipoprotein(a) level (Hollstein et al., 2019; Sabatine, 2019). However, the clinical relevance of these different metabolic changes is uncertain and difficult to assess. In the large outcome studies discussed below, statins and PCSK9 inhibitors were administered simultaneously in the majority of study participants (Sabatine et al., 2017a; Schwartz et al., 2018).

The metabolic changes induced by another way of pharmacologic inhibition of PCSK9, i.e., with the small interfering RNA inclisiran, are similar to those observed under treatment with antibodies against PCSK9 (Raal et al., 2020).

The therapeutic inhibition of PCSK9 may have beneficial effects beyond LDL-C metabolism and atherosclerosis. In general, implications of PCSK9 in inflammation, blood pressure regulation, glucose metabolism, and adipogenesis have been described (Urban et al., 2013). This may explain data that indicate beneficial effects of PCSK9 inhibition on non-alcoholic fatty liver disease (Theocharidou et al., 2018). Furthermore, as PCSK9 was first discovered in brain and in patients with Alzheimer’s disease, high concentrations of PCSK9 in cerebrospinal fluid have been found, a role of PCSK9 in Alzheimer’s disease has been controversially discussed (Adorni et al., 2019). Future studies and long-term follow-up data of clinical trials may help to clarify whether PCSK9 inhibition may be a valuable therapeutic option in these conditions.

## LDL-C Lowering With PCSK9 Antibodies

Many approaches to inhibit PCKS9 pharmacologically are being pursued. Clearly, the most advanced of those is the use of antibodies. Initially, three antibodies have been tested in clinical programs. While evolocumab and alirocumab are fully human antibodies, bococizumab is a humanized, but not fully human antibody. At later stages of development, neutralizing antibodies against bococizumab were detected, which impaired the long-term effects of the drug on LDL-C and led to the discontinuation of its further development. The following section therefore focusses on data of the currently available PCSK9 antibodies evolocumab and alirocumab.

Both evolocumab and alirocumab are administered subcutaneously. While evolocumab was applied at a dose of 140 mg every 2 weeks or 420 mg every month in all phase 3 studies, alirocumab, in most instances, was used at a dose of 75 mg every 2 weeks, which was increased to 150 mg every 2 weeks if LDL-C level remained greater than 70 mg/dL; alternative dose regimens included 300 mg every 4 weeks and 150 mg every 2 weeks without previous lower dosing (Robinson et al., 2015; Roth et al., 2016; Scherer et al., 2017; Blom et al., 2020).

Apart from the large cardiovascular outcome studies, both evolocumab and alirocumab were tested in several clinical settings, including monotherapy, statin-treated patients, heterozygous and homozygous familial hypercholesterolemia (HeFH and HoFH), and statin-intolerant patients. The results of selected key studies are summarized in Table 1 and described in the following section.

**TABLE 1:** Key studies on LDL cholesterol reduction with PCSK9 antibodies.

**Population/therapeutic regimen**	**Drug**	**Study**	***n***	**Duration (weeks or as indicated)**	**Baseline LDL-C**	**Mean LDL-C reduction compared to placebo**
PCSK9 antibody monotherapy	Evolocumab	MENDEL-2 (Koren et al., 2014)	614	12	143 mg/dL	55–57%
	Alirocumab	ODYSSEY MONO (Roth et al., 2014)	103	24	140 mg/dL	47%^†^
PCSK9 antibody in addition to statin	Evolocumab	LAPLACE-2 (Robinson et al., 2014)	1,896	12	109 mg/dL	63–75%
		DESCARTES (Blom et al., 2014)	901	52	104 mg/dL	57%
		GLAGOV (Nicholls et al., 2016)	968	76	93 mg/dL	61%
		EVOPACS (Koskinas et al., 2019)	308	8	136 mg/dL	41%
	Alirocumab	ODYSSEY COMBO-I (Kereiakes et al., 2015)	316	24	97 mg/dL	46%
		ODYSSEY COMBO-II (Cannon et al., 2015)	720	104	107 mg/dL	51%^†^ (week 24)
		ODYSSEY CHOICE I (Roth et al., 2016)*	803 (547 with statin)	24	116 mg/dL (patients with statin)	59%
		ODYSSEY LONG TERM (Robinson et al., 2015)#	2,341	78	122 mg/dL	62%
Statin-intolerant patients	Evolocumab	GAUSS-2 (Stroes et al., 2014)	307	12	193 mg/dL	55–56%^†^
		GAUSS-3 (Nissen et al., 2016)	511 (218 randomized)	24	220 mg/dL	53%^†^
	Alirocumab	ODYSSEY ALTERNATIVE (Moriarty et al., 2015)	361 (314 randomized)	24	191 mg/dL	45%^†^
Familial hypercholesterolemia	Evolocumab	RUTHERFORD-2 (HeFH) (Raal et al., 2015b)	329	12	155 mg/dL	59–61%
		TESLA Part B (HoFH) (Raal et al., 2015a)	50	12	348 mg/dL	31%
		TAUSSIG (HoFH) (Raal et al., 2017)	106	1.7 years	326 mg/dL	21%^†^ (week 12)
		HAUSER-RCT (pediatric HeFH) (Santos et al., 2020a)	157	24	184 mg/dL	38%
	Alirocumab	ODYSSEY FH 1/FH 2 (HeFH) (Kastelein et al., 2015)	735 (486/249)	78	145/135 mg/dL	58/51% (week 24)
		HoFH, compound/double heterozygous FH (Hartgers et al., 2018)	20	≥12	194 mg/dL	9–65%^†^ (week 24)
		ODYSSEY HoFH (Blom et al., 2020)^#^	69	12	283 mg/dL	36%

*Evolocumab was administered at doses of 140 mg every 2 weeks or 420 mg monthly, alirocumab at 75 mg every 2 weeks and increased to 150 mg if LDL-C was >70 mg/dL, or as indicated by ^∗^(300 mg every 4 weeks) and ^#^(150 mg every 2 weeks). ^†^not placebo-controlled.Abbreviations: ASCVD, atherosclerotic cardiovascular disease, HeFH, heterozygous familial hypercholesterolemia, HoFH, homozygous familial hypercholesterolemia.*

### Monotherapy

*Evolocumab* was tested as monotherapy in 614 patients with hypercholesterolemia defined as LDL-C ≥100 and <190 mg/dL. Patients were not allowed to be on lipid-lowering therapies, and patients with CAD, diabetes or other medical conditions were excluded. Participants were randomly assigned to ezetimibe or placebo (oral) and evolocumab or placebo biweekly or monthly (subcutaneous). Compared to placebo, biweekly and monthly administered evolocumab reduced LDL-C from a mean baseline of 143 mg/dL by 57 and 55% after 12 weeks, respectively (Koren et al., 2014).

*Alirocumab* was tested as monotherapy in 103 patients with moderately elevated cardiovascular risk who did not take any lipid-lowering therapy. LDL-C had to be between 100 and 190 mg/dL. Patients were treated with alirocumab or matching placebo. Alirocumab was administered at a dose of 75 mg biweekly. If LDL-C was ≥70 mg/dL at week 8, the dose was increased to 150 mg biweekly at week 12. After 24 weeks, LDL-C was reduced by 47% (intention-to-treat) and 53% (on-treatment) (Roth et al., 2014). Furthermore, in a subgroup of a study that tested the dose of 300 mg of alirocumab every 4 weeks (ODYSSEY CHOICE I), participants without statin showed a LDL-C reduction of 52% at week 24 (Roth et al., 2016).

### Addition to Statins

Statin therapy is without doubt the cornerstone of any LDL-C-lowering therapy. Therefore, any novel LDL-C-lowering medication would need to show efficacy on top of statin therapy or only be indicated in the minority of patients not tolerating statins.

*Evolocumab*. In the LAPLACE-2 study, evolocumab was tested against placebo in patients with hypercholesterolemia (LDL-C >150 mg/dL if untreated, or >80–100 mg/dL depending on statin intensity) (Robinson et al., 2014). In two steps of a complex randomization procedure, first, all participants were randomized to different regimens of moderate- or high-intensity statin therapy with different statins, after which mean LDL-C was 109 mg/dL – lower in the group with high-intensity statin, and higher in the group with moderate-intensity statin. In the second step, the 1,899 participants were randomized (1,896 received the study drug) to evolocumab biweekly (140 mg) or monthly (420 mg) and additionally, in the patients treated with atorvastatin, to ezetimibe or placebo. Evolocumab lowered LDL-C in all groups of statin therapy with no signs of interaction by drug or intensity of therapy by 66–75% (biweekly administration) and 63–75% (monthly administration) compared to placebo.

In the DESCARTES study, 901 patients with hypercholesterolemia were included (Blom et al., 2014). Based on cardiovascular risk and baseline LDL-C, patients were assigned to diet or atorvastatin at doses of up to 80 mg plus optionally ezetimibe. After this run-in period of 4–12 weeks, patients with LDL-C ≥75 mg/dL were randomly assigned to evolocumab 420 mg monthly or placebo in a 2:1 ratio. Baseline LDL-C, after the run-in period, was 104 mg/dL; 15% of patients had CAD. At week 52, LDL-C was reduced by 57% compared to placebo.

The objective of the GLAGOV study was to examine whether PCSK9 inhibition might affect coronary plaque progression (Nicholls et al., 2016). The study included 968 patients with angiographically documented CAD. Mean baseline LDL-C was 93 mg/dL, almost all patients were treated with a statin. Randomization was 1:1 to evolocumab 420 mg or placebo monthly for 78 weeks. At follow-up, patients underwent serial coronary angiography. Imaging with intravascular ultrasonography revealed significant reductions in atheroma volume in patients on evolocumab, but not on placebo. A larger proportion of patients under evolocumab compared to placebo showed plaque regression (64 vs. 47%). Although not a cardiovascular outcome study, the GLAGOV trial was an important step in suggesting a potential pathophysiologic cascade from reducing LDL-C to eventually reducing cardiovascular events via reduction of atherosclerotic burden.

The EVOPACS study was an investigator-initiated study of evolocumab in the setting of acute coronary syndrome (Koskinas et al., 2019). 308 patients hospitalized for acute coronary syndrome were included if their LDL-C was ≥70 mg/dL despite high-intensity statin therapy for 4 weeks, ≥90 mg/dL despite low- or moderate-intensity statin, or ≥125 mg/dL with no statin pre-treatment. Randomization was 1:1 to evolocumab 420 mg every 4 weeks or placebo in addition to high-intensity statin therapy. Most patients (78%) were not on statin at baseline. In both the evolocumab and the placebo group, LDL-C was reduced at week 8 (evolocumab: 140 mg/dL to 31 mg/dL, –77%, placebo: 132 mg/dL to 80 mg/dL, –35%). The difference in mean LDL-C reduction was 40.7%. In the evolocumab group, 95.7% achieved LDL-C levels <70 mg/dL as compared to 37.6% in the placebo group. Adverse and cardiovascular events were similar in both groups. This study was the first to show safety of initiating PCSK9 inhibitor treatment during hospitalization for acute coronary syndrome.

*Alirocumab*. In the ODYSSEY COMBO I study, alirocumab was tested on top of maximally tolerated statin therapy in 316 patients with CAD or risk equivalents (Kereiakes et al., 2015). As in previous studies, alirocumab dose was increased from 75 to 150 mg biweekly at week 12 if the 8-week LDL-C was ≥70 mg/dL. Mean baseline LDL-C was 97 mg/dL. Patients were randomized to alirocumab or placebo in a 2:1 ratio. Alirocumab reduced LDL-C at week 24 by 45.9% compared to placebo. The successor study ODYSSEY COMBO II included 720 patients with similar characteristics (Cannon et al., 2015). The patients were randomized to alirocumab or ezetimibe (2:1) and respective placebos. At week 24, LDL-C was reduced by 51% from a baseline mean of 107 mg/dL, which was maintained until week 52 (–50% under alirocumab with blinded dose adjustments as described previously, and –18% under ezetimibe).

In the previously mentioned ODYSSEY CHOICE I study, patients without and with statin therapy were included (Roth et al., 2016). Differently from other studies with alirocumab, a dose of 300 mg every 4 weeks was administered. Participants had elevated cardiovascular risk and hypercholesterolemia. At week 24, LDL-C was reduced by 59% compared to placebo from a mean baseline LDL-C of 116 mg/dL.

In the ODYSSEY LONG TERM study, 2,341 patients at high risk for cardiovascular events with an LDL-C of ≥70 mg/dL were randomized to receive alirocumab 150 mg biweekly or placebo in a 2:1 fashion (Robinson et al., 2015). Patients had to be on maximally tolerated statin therapy. Mean baseline LDL-C was 122 mg/dL, 69% had CAD. Compared to placebo, alirocumab reduced LDL-C by 62% at week 24, with this effect being maintained throughout week 78 (–56%). Adverse events and serious adverse events were not statistically different between alirocumab and placebo. Patients under alirocumab more often had injection-site reactions and myalgia. Major adverse cardiovascular events occurred in fewer patients under alirocumab compared to placebo, although this analysis was *post hoc*.

In summary, the study results described above show that treatment with PCSK9 antibodies results in similar reductions of LDL-C regardless of statin treatment, statin type, or statin intensity, even if statin therapy has been shown to induce the expression of PCSK9 (Dubuc et al., 2004; Silbernagel et al., 2019).

### Patients With Statin-Associated Muscle Symptoms

Statin-associated muscle symptoms are among the most frequently reported side effects of statin therapy, although in randomized trials, they occur in comparable frequencies under statin and placebo (Laufs and Isermann, 2020). However, although a causal association between statin intake and myalgia cannot always be established, it often leads to low adherence and discontinuation of statin therapy which in turn contributes to adverse cardiovascular outcomes (Stroes et al., 2015). Therapeutic approaches to lower LDL-C other than statins therefore play an important role in clinical practice. Real-world data from Germany underline the importance of antibodies against PCSK9 in this setting by showing that among patients treated with alirocumab, 73% had partial or total statin intolerance (Parhofer et al., 2019).

The safety and efficacy of both evolocumab and alirocumab has been investigated in the setting of statin intolerance.

*Evolocumab* was tested in 307 patients with intolerance of effective doses of at least 2 different statins in the GAUSS-2 study (Stroes et al., 2014). Low-dose statins were allowed. Evolocumab was compared to ezetimibe. From a mean baseline LDL-C of 193 mg/dL, evolocumab reduced LDL-C by 55–56%, while muscle adverse events occurred in 12% of patients on evolocumab, and 23% on ezetimibe. The GAUSS-3 study initially included 511 patients with intolerance of at least 2 statins (Nissen et al., 2016). In a first step, patients were randomized to atorvastatin or placebo in a crossover procedure to identify those patients with muscle symptoms under statin but with no symptoms under placebo. This criterion was met by 43% of patients. They subsequently entered, alongside a few patients with elevated creatine kinase, the next step in which these 218 patients were randomized to evolocumab or ezetimibe (2:1). At week 24, evolocumab reduced LDL-C by 53%. The frequency of muscle symptoms was not significantly different between evolocumab and ezetimibe (21 and 29% respectively).

*Alirocumab* was investigated in statin-intolerant patients in the ODYSSEY ALTERNATIVE study. Patients with intolerance against at least two statins were included (Moriarty et al., 2015). Of the initial 361 participants, only those who did not report muscle symptoms in a placebo run-in phase of 4 weeks (oral and subcutaneous placebo) continued the study. These 314 patients were randomized to alirocumab, ezetimibe, or atorvastatin and oral or subcutaneous placebo, respectively. Alirocumab dose was increased at week 12 as described previously. The mean baseline LDL-C was 193 mg/dL. Alirocumab reduced LDL-C by 45% at week 24. Muscle symptoms under alirocumab occurred significantly less often than under atorvastatin (hazard ratio 0.61 [95% CI, 0.38–0.99]).

### Familial Hypercholesterolemia (FH)

Another population in which statins are often not sufficiently efficacious are patients with FH. As discussed, the mutations found in these patients lead to dysfunctional LDL receptors (mutations in *LDLR*), diminish interaction of LDL particles with the LDL receptor (mutations in *APOB*), or decrease LDL receptor recycling (gain-of-function mutations in *PCSK9*). As the final step of the mechanism of action of statins and ezetimibe is the upregulation of the LDL receptor, and the pathophysiology of FH involves this particular receptor, patients with FH do not only have markedly elevated LDL-C, but quite often are also difficult to treat. Fortunately, in patients with the more common heterozygous FH (HeFH) who have at least one normal *LDLR* allele and therefore, residual functionality of the LDL receptor pathway, PCSK9 antibodies still cause marked reductions in LDL-C, comparable to the reductions observed in patients without proven HeFH. In contrast, in the rare homozygous FH (HoFH), the efficacy of PCSK9 antibodies depends on the mutations these patients have and their ability of upregulating the LDL receptor.

*Evolocumab*. The RUTHERFORD-2 study included 331 patients who met the Simon Broome criteria of HeFH, for which genetic testing is not necessarily required (Raal et al., 2015b). Of 264 patients who consented to genetic testing, 211 (80%) had a mutations causative of FH. LDL-C had to be ≥100 mg/dL despite stable lipid-lowering therapy. Mean baseline LDL-C was 155 mg/dL, 31% of the patients had CAD. Patients were randomized to evolocumab or placebo in a 2:1 fashion, 329 received at least one dose of either evolocumab or placebo. Compared to placebo, evolocumab reduced LDL-C by 59–61% at week 12. Patient response to evolocumab was independent of the type of mutation present.

In the TESLA Part B study, the effect of evolocumab on LDL-C in patients with HoFH was investigated (Raal et al., 2015a). HoFH was either diagnosed by genetic testing or clinically, defined as an untreated LDL-C >503 mg/dL and either xanthomas before 10 years of age or evidence of HeFH in both parents. LDL-C had to be ≥131 mg/dL. Fifty patients with HoFH and not on lipoprotein apheresis were included. The mean age was 31 years, 43% had CAD, and 25% had undergone cardiac bypass surgery. Mean baseline LDL-C was 348 mg/dL. HoFH was genetically confirmed in all patients except for one participant with clinical HoFH, but genetically confirmed HeFH. The causal mutations were mainly located in the *LDLR* gene, with similar proportions of true homozygous and compound heterozygous mutation carriers. The patients were randomized to 420 mg evolocumab every 4 weeks or placebo in a 2:1 fashion. At week 12, evolocumab reduced LDL-C by 31% compared to placebo. LDL-C reduction was 41% in patients with at least one defective LDL receptor allele, 47% in patients with 2 defective alleles, 25% in defective/negative LDL receptor status, and 22% with unclassified mutation status. In the one patient with negative/negative LDL receptor status, LDL-C increased by 10% under evolocumab. While there were marked differences in response to evolocumab among the different mutations, also patients carrying the same mutation showed variable treatment response. For example, 8 patients with the same LDL receptor mutation had LDL-C reductions ranging between 7 and 56% with evolocumab, suggesting that mechanisms other than the underlying mutations must be involved in determining the response to treatment.

The TAUSSIG study was another investigation of patients with HoFH with similar inclusion criteria as the TESLA Part B study. Patients included in the TESLA studies could enter the open-label TAUSSIG study. Patients received evolocumab 420 mg every 4 weeks or every 2 weeks if they were on lipoprotein apheresis (Raal et al., 2017). In patients not on apheresis, the dose could be increased to biweekly administration of 420 mg evolocumab after 12 weeks. A hundred and six patients were enrolled, of whom 34 received apheresis. Baseline LDL-C was 326 mg/dL. At week 12, LDL-C was decreased from baseline by 20.6%, which was maintained until week 48. Doubling the dose of evolocumab in patients not receiving apheresis led to an additional 8% LDL-C decrease. LDL-C was reduced both in patients receiving apheresis and those who did not. The follow-up was 1.7 years, during which the therapy was tolerated well. The report of long-term data of the TAUSSIG study additionally included patients with severe HeFH, which was determined on the investigator’s discretion based on response to treatment, presence of ASCVD, and cardiovascular risk factors (Santos et al., 2020b). 300 patients, of which 106 had HoFH, were treated with evolocumab over a median 4.1 years. Mean LDL-C change from baseline was 21.2% in HoFH patients and 54.9% in HeFH patients. Up-titration of evolocumab in HoFH patients not on apheresis increased the LDL-C reduction from 19.6 to 29.7% from baseline. Sixteen out of 61 patients on apheresis at baseline were able to discontinue apheresis. The most common adverse events included nasopharyngitis, influenza, upper respiratory tract infection, and headache. The reductions in LDL-C were maintained over time.

The HAUSER study (Santos et al., 2020a) included pediatric patients aged 10–17 years with HeFH with an LDL-C ≥130 mg/dL on stable lipid-lowering therapy. One hundred and fifty-seven patients were randomized to evolocumab 420 mg monthly or placebo in a 2:1 fashion. The mean age was 14 years, mean baseline LDL-C was 184 mg/dL. The majority of mutations were located in the *LDLR* gene. After 24 weeks of treatment, evolocumab reduced LDL-C by 38.3% compared to placebo, corresponding to an absolute reduction of 68.6 mg/dL. Adverse events occurred in a similar proportion under evolocumab and placebo. Although the time of follow-up was limited, especially considering the potential life-long treatment for this genetic disorder, this study provides valuable information for this population at high lifetime risk of ASCVD.

*Alirocumab* was tested in HeFH patients in the ODYSSEY FH I and FH II study (Kastelein et al., 2015). The studies included a total of 735 patients with HeFH, diagnosed by genetic testing or clinically according to the Simon Broome or Dutch Lipid Network criteria. LDL-C had to be ≥100 mg/dL in patients without and ≥70 mg/dL with previous cardiovascular event. All patients were required to receive high-dose statins unless not tolerated. Patients were randomized in a 2:1 ratio to receive alirocumab 75 mg or placebo every 2 weeks. The dose was increased to 150 mg if LDL-C was ≥70 mg/dL at week 8. At week 24, the mean baseline LDL-C of 145 and 135 mg/dL (FH I and FH II) had decreased by 57.9 and 51.4% as compared with placebo. These changes were maintained in week 78.

In an analysis of different placebo-controlled trials with alirocumab, the effects of alirocumab in patients with HoFH, double heterozygous FH (mutations in two different genes), and compound heterozygous FH (different mutations in both alleles of the same gene) were investigated (Hartgers et al., 2018). Patients with a diagnosis of FH and elevated LDL-C from 6 different alirocumab trials were sequenced and included if they had two FH-causing mutations. A total of 20 patients qualified for the study, of which 7 were double heterozygotes, 10 compound heterozygotes, and 3 had HoFH. Eleven patients received alirocumab 75 mg every 2 weeks (increased to 150 mg as previously described) or 150 mg biweekly from the beginning on, and 9 received placebo. Baseline LDL-C was 194 mg/dL. After 12 or 24 weeks of treatment, LDL-C reductions of at least 15% were observed in all patients. At week 24, the reductions in LDL-C ranged between 9 and 65%.

The ODYSSEY HoFH trial is the largest randomized controlled interventional trial in HoFH to date (Blom et al., 2020). The trial enrolled patients that were at least 18 years of age, had a clinical or genetic diagnosis of HoFH, and an LDL-C of ≥70 mg/dL. For patients on lipoprotein apheresis, genetic confirmation of HoFH was mandatory. To be eligible for inclusion, patients were required to be on stable statin therapy or have statin intolerance. The patients were randomized in a 2:1 fashion to receive biweekly alirocumab 150 mg or placebo during 12 weeks of treatment, which was followed by a 12-week open-label treatment period with all participants receiving alirocumab. A total of 69 patients were included, of which 10 were on lipoprotein apheresis. The mean age was 42.3 and 45.4 years in the alirocumab and placebo group, respectively, 46.7 and 37.5% of patients had a history of CAD. Most patients (∼ 41%) had a homozygous mutation in the *LDLR* gene. Mean baseline LDL-C was 295 and 260 mg/dL in the alirocumab and placebo group, respectively. At week 12, alirocumab reduced LDL-C compared to placebo by 35.6%. As with evolocumab, the efficacy of alirocumab to lower LDL-C was highly variable among participants with negative/negative LDL receptor status. Treatment with alirocumab was well tolerated.

Based on the available data, both evolocumab and alirocumab are approved for HeFH, but to date only evolocumab is approved for HoFH.

### Long-Term Effects on LDL-C

Patients enrolled in any one of the 12 studies of the evolocumab study program could be included in the open-label extension studies OSLER-1 (phase 2 studies) and OSLER-2 (phase 3 studies) (Sabatine et al., 2015). A total of 4,465 patients provided their consent to participate in the open-lapel extension. Their baseline characteristics depended on the inclusion criteria of the parent trial. Patients were randomized to receive evolocumab 140 mg every 2 weeks or 420 mg every month plus standard care or standard care alone in a 2:1 ratio, irrespective of treatment allocation in the parent trial. Follow-up was 11 months. From a median baseline LDL-C of 120 mg/dL, evolocumab reduced LDL-C by 61% compared to standard care. Neurocognitive events were more often reported in the evolocumab group (0.9 vs. 0.3%), but did not vary depending on the achieved LDL-C level. Cardiovascular events were numerically lower in the evolocumab-treated patients (0.95 vs. 2.18%).

The OSLER-1 study continued further. After the additional 1-year follow-up with repeat randomization, patients could enter the all-evolocumab period with additional 4-year follow-up (Koren et al., 2019). 1,151 patients were enrolled. Evolocumab persistently lowered LDL-C by ∼56% at 5-year follow-up. Serious adverse events occurred in 6.9–7.9% of patients per year, comparable to 6.8% in the standard care group in the first year. 4 patients developed binding anti-drug antibodies, which were transient and did not impair efficacy of treatment, and no patient developed drug-neutralizing antibodies. This study represents the longest follow-up of PCSK9 antibody treatment to date, with maintained efficacy in lowering of LDL-C, good tolerability (discontinuation 5.7%), and safety.

Evidence of sustained reductions in LDL-C by treatment with alirocumab derives from a subgroup analysis of the ODYSSEY Outcomes study (Schwartz et al., 2018) which is discussed below in detail. In this subgroup analysis (Goodman et al., 2020), *n* = 3,551 patients (18.8% of the total ODYSSEY Outcomes cohort) were investigated. All these patients had follow-up of at least 3 years, had no missing LDL-C measurements, no change in background lipid-lowering therapy, no discontinuation of study treatment, and no blinded substitution of alirocumab with placebo due to LDL-C values <15 mg/dL. Over 3 years, no attenuation in efficacy of alirocumab to lower LDL-C was observed.

### Cardiovascular Outcome Studies With PCSK9 Antibodies

#### SPIRE Studies

The publication of the SPIRE I and II trials (Ridker et al., 2017a) is the first report on the efficacy of a PCSK9 antibody to not only lower LDL-C, but also the risk of recurrent cardiovascular events in patients with or at high risk for ASCVD.

The SPIRE I and II trials included a total of 27,438 patients with LDL-C levels of at least 70 mg/dL (SPIRE I) and 100 mg/dL (SPIRE II), respectively. Patients were eligible for inclusion if they were at high risk for ASCVD without previous event, or if they already had an ASCVD event. Treatment with high-intensity statin therapy was mandatory.

Participants received 150 mg of bococizumab subcutaneously every 2 weeks. Under treatment, LDL-C was reduced by 59% at week 14 compared to placebo. The primary endpoint – a composite of myocardial infarction, stroke, hospitalization for unstable angina requiring urgent revascularization, and cardiovascular death – was not significantly reduced in SPIRE I (lower cardiovascular risk, follow-up 7 months) with a hazard ratio of 0.99 (95% CI, 0.80–1.22), but was significantly reduced in SPIRE II (higher cardiovascular risk, follow-up 12 months) with a hazard ratio of 0.79 (95% CI, 0.65–0.97). Injection-site reactions were markedly more common with bococizumab than with placebo (10.4 vs. 1.3%).

Both trials were prematurely terminated, as in other studies of the SPIRE program, nearly half the patients developed antibodies against bococizumab which attenuated the magnitude and durability of LDL-C lowering (Ridker et al., 2017b). Importantly, the relative risk reduction in the SPIRE studies correlated with the achieved reductions in LDL-C, confirming the therapeutic principle of PCSK9 inhibition. The clinical development of bococizumab was terminated.

#### FOURIER Trial

In the landmark FOURIER trial (Sabatine et al., 2017a), the PCSK9 antibody evolocumab was shown to effectively lower LDL-C and the risk of recurrent cardiovascular events in patients with established ASCVD (Table 2).

**TABLE 2:** Comparison of the FOURIER and ODYSSEY Outcomes studies.

	**FOURIER** (Sabatine et al., 2017a)	**ODYSSEY Outcomes** (Schwartz et al., 2018)
Inclusion criteria	• Myocardial infarction, stroke, or peripheral artery disease and • LDL-C >70 mg/dL under statin therapy	• Acute coronary syndrome 1–12 months ago and • LDL-C >70 mg/dL under statin therapy
*n* =	• 27,564	• 18,924
Drug	• Evolocumab 140 mg every 2 weeks or 420 mg monthly	• Alirocumab 75 or 150 mg every 2 weeks • LDL-C target 25−50 mg/dL
LDL-C after 1 year of treatment	• 30 mg/dL	∙ 48 mg/dL
Primary endpoint	Composite of • cardiovascular death • myocardial infarction • non-hemorrhagic stroke • hospitalization for unstable angina • coronary revascularization	Composite of • coronary death • myocardial infarction • ischemic stroke • hospitalization for unstable angina
Median follow-up	• 2.2 years	• 2.8 years
Hazard ratio (95% confidence interval) for the primary endpoint	• 0.85 (0.79–0.92)	• 0.85 (0.78–0.93)

*Abbreviation: LDL-C, low-density lipoprotein cholesterol.*

##### Study population, treatment protocol, endpoints

Patients were eligible to be included in the FOURIER study if they had a history of ASCVD defined as myocardial infarction, non-hemorrhagic stroke, or symptomatic peripheral artery disease, each with additional cardiovascular risk factors. Patients were required to be on optimized statin therapy with an equivalent dose of at least atorvastatin 20 mg, and an LDL-C of greater than 70 mg/dL. Patients were randomized to either evolocumab (subcutaneous doses of 140 mg every 2 weeks or 420 mg monthly, according to patient preference) or placebo in a 1:1 fashion. The primary endpoint was a composite of cardiovascular death, myocardial infarction, stroke, hospitalization for unstable angina, or coronary revascularization.

##### Results

A total of 27,564 patients with a mean age of 62.5 years (25% women) were randomized to evolocumab or placebo. Among the participants, 81% had a history of myocardial infarction, 19% of non-hemorrhagic stroke, and 13% had symptomatic peripheral artery disease. Diabetes mellitus was present in 37% of the patients, hypertension in 80%, and 28% were current smokers. High-intensity statin therapy was established in 69% of the patients. Median baseline LDL-C was 92 mg/dL.

After 48 weeks of treatment, LDL-C was reduced by 59% in patients on evolocumab as compared to placebo, corresponding to an absolute reduction of 62 mg/dL down to a remarkably low median LDL-C of 30 mg/dL. This reduction was maintained throughout follow-up. Similar relative reductions were also observed for non-HDL cholesterol and apolipoprotein B.

Evolocumab reduced the relative risk of the primary endpoint by 15% with a hazard ratio of 0.85 (95% CI, 0.79–0.92) during a median follow-up of 2.2 years. The risk of the key secondary endpoint, a composite of cardiovascular death, myocardial infarction, or stroke, had a relative reduction of 20%. While among the endpoint components, the risk of myocardial infarction, stroke, and coronary revascularization were significantly reduced, there were no significant reductions in the risk of unstable angina, and, importantly, cardiovascular or all-cause death. The reduction in risk increased with time. The results were consistent in all subgroups independent of age, gender, ASCVD type, baseline LDL-C, and statin intensity.

Adverse events occurred in similar frequencies in the evolocumab and placebo group. Injection-site reactions were slightly more common with evolocumab (2.1 vs. 1.6%), but were classified as mild in both groups in 90% of the cases and led to discontinuation of treatment or placebo in only 0.1%, with no significant differences between groups.

##### Secondary and post hoc analyses

Several secondary and *post hoc* analyses have been conducted with data of the FOURIER study.

*Interindividual response to treatment*. Although the mean reduction in LDL-C under evolocumab is remarkable, the possibility existed that some patients would not respond to treatment, and others would respond better than average. Therefore, an analysis was conducted in which the interindividual variation in response to evolocumab treatment was investigated. The authors found a highly consistent reduction in LDL-C, with 94.7% of patients having reductions in LDL-C of 50% or more during the first year, 97.9% having reductions of 30% or more, and only 0.5% of patients having no apparent reduction in LDL-C level in response to treatment (Qamar et al., 2018).

*Subgroups*. Patients of different subgroups generally gained comparable benefits in terms of relative risk reductions. Patients with higher cardiovascular risk due to the presence of cardiovascular risk factors or more severe atherosclerotic disease had the largest clinical benefit as measured by the absolute risk reduction (or its reciprocal, the number needed to treat).

This notion in general is supported by an analysis including clinical risk factors and genetic risk, the latter based on a genetic risk score including 27 single nucleotide polymorphisms. Patients who had no clinical risk factors or no elevated genetic risk had low event rates. With increasing clinical and/or genetic risk, event rates increased. The absolute benefits of treatment with evolocumab were found to be greatest in the group with the highest genetic risk (Marston et al., 2020b).

At the time the FOURIER study was published, guidelines on dyslipidemia recommended LDL-C goals of <70 mg/dL for patients with ASCVD (Catapano et al., 2016). It was uncertain whether patients would benefit from lowering LDL-C below this target value. In a secondary analysis, comparing patients with baseline LDL-C values below and over 70 mg/dL, the cardiovascular benefits from evolocumab treatment were similar in both groups. Furthermore, patients benefitted regardless of receiving statin therapy of maximal or submaximal potency (Giugliano et al., 2017a).

Benefits with respect to the primary and secondary endpoints were also found in patients with and without type 2 diabetes at baseline. Interestingly, evolocumab did not result in an increased risk of developing diabetes, or worsening glycemia (Sabatine et al., 2017b). This is in contrast to what genetic studies suggested (Ference et al., 2016; Schmidt et al., 2017). Explanations for this discrepancy include the short trial duration, and the possibility that the risk of new-onset diabetes depends on an intracellular reduction of PCSK9 (which results from genetic variation in the *PCSK9* gene, but not from treatment with antibodies binding only extracellular PCSK9). Longer follow-up data may potentially contribute to a better understanding of this issue and its clinical relevance.

With respect to previous myocardial infarction, patients closer to the most recent event, a history of multiple myocardial infarctions, or multivessel CAD tended to have greater relative risk reductions from evolocumab treatment than patients without. The corresponding absolute risk reduction in this groups were substantially greater (∼3.5%, compared to ∼1%) (Sabatine et al., 2018). Furthermore, patients with recent myocardial infarction (within 12 months prior to randomization) were at higher risk compared to patients with a remote event (more than 12 months prior to randomization), and gained greater absolute benefit from evolocumab (Gencer et al., 2020b). Evolocumab reduced the risk of myocardial infarctions related to plaque rupture, did not reduce the risk of type 2 infarctions (induced by a mismatch of oxygen demand and supply but without atherothrombotic occlusion of a coronary artery), and reduced the risk in a similar fashion for small vs. large infarctions and infarctions with or without ST-segment elevation (Wiviott et al., 2020).

Patients with peripheral artery disease (*n* = 3642) had a greater risk of experiencing a primary endpoint event, gained comparable relative and remarkably greater absolute benefits compared to patients without peripheral artery disease (absolute risk reduction 3.5 vs. 1.6%). The risk of major adverse limb events – defined as acute limb ischemia, major amputation, or urgent peripheral revascularization because of ischemia – was significantly reduced by evolocumab treatment (Bonaca et al., 2018). This secondary analysis provides crucial information for the treatment of patients with peripheral artery disease and underlines the high cardiovascular risk these patients are exposed to.

In the FOURIER study, 59.8% of the patients had a metabolic syndrome according to a definition based on waist circumference, blood pressure, and levels of triglycerides, HDL cholesterol, and fasting glucose (Grundy et al., 2005). Patients with metabolic syndrome had a higher event rate than patients without (Deedwania et al., 2020). The reductions in LDL-C level were comparable in patients with and without metabolic syndrome, as were the relative reductions in risk for the primary and key secondary endpoints. In the subgroup of patients with metabolic syndrome, the risk of new-onset diabetes or worsening glycemic control was not affected by treatment with evolocumab.

Finally, evolocumab reduced the primary endpoint and the key secondary endpoint in men and women without significant differences, and in a comparable magnitude in patients stratified by quartiles of age (Sever et al., 2020). The risk of adverse events was higher in older patients and in women, but apart from injection-site reactions, there were no important significant differences.

*Stroke*. With respect to the prevention of stroke, evolocumab significantly reduced the risk of all strokes (1.5 vs. 1.9%), and the risk of ischemic stroke (1.2 vs. 1.6%). The risk of hemorrhagic stroke remained unaffected. These findings were consistent in subgroup analyses of patients with prior ischemic stroke (19%) compared to patients without (Giugliano et al., 2020).

*Inflammation and chronic kidney disease* are now established independent cardiovascular risk factors (Ridker et al., 2008; Gansevoort et al., 2013). Regarding the former, a secondary analysis confirmed that patients in the highest tertile of CRP level in the placebo arm had a higher risk of primary endpoint events (12.0% in the lowest compared to 18% in the highest tertile). The relative risk reduction was comparable among the tertiles, and correspondingly, the absolute risk reduction was greatest in the highest CRP tertile (Bohula et al., 2018). Regarding chronic kidney disease, it was found that when comparing chronic kidney disease stages 1, 2, and 3 or greater, the efficacy of evolocumab to lower LDL-C and the relative risk reductions were comparable among the categories. Because of the higher baseline risk associated with higher degree of kidney dysfunction, the absolute benefits were greatest in the group with chronic kidney disease stage 3 or greater (Charytan et al., 2019).

*Lipoprotein(a)*. Another important and interesting aspect of PCSK9 inhibition is the reduction of lipoprotein(a), an independent cardiovascular risk factor, by 20–30%. The mechanisms by which PCSK9 antibodies reduce lipoprotein(a) may involve LDL receptor-mediated pathways (Raal et al., 2016), but are still not completely understood. Interestingly, as opposed to most LDL-C-lowering therapies, PCSK9 antibodies do not lower lipoprotein(a) proportionally through the whole range of lipoprotein(a) levels: The relative reduction diminishes with higher lipoprotein(a) levels, possibly due to reduced clearance of lipoprotein(a) particles with small isoform size (O’Donoghue et al., 2018). In FOURIER, lipoprotein(a) was reduced by a median of 26.9% after treatment with evolocumab. In the placebo arm, patients in the highest lipoprotein(a) quartile had a significantly increased cardiovascular risk compared to patients in the first quartile, independently of LDL-C concentrations. Comparing patients with lipoprotein(a) level above vs. below the median, the relative risk reduction was 23 and 7%, respectively (*p* for interaction, 0.07). The absolute risk reduction was therefore considerably larger in patients with lipoprotein(a) above the median (2.49 vs. 0.95%) (O’Donoghue et al., 2018). However, it is not possible to finally determine which proportion of cardiovascular risk reduction by treatment with evolocumab is attributable to the reduction of lipoprotein(a) or LDL-C based on this analysis. Another interesting *post hoc* analysis shows that evolocumab also reduced the risk of venous thromboembolic events, and suggests that the magnitude of risk reduction may be determined by the reduction of lipoprotein(a) – a potentially important suggestion given the ongoing outcomes trials on specific and potent lowering of lipoprotein(a) with antisense oligonucleotides (NCT04023552) (ClinicalTrials.gov, 2020b; Marston et al., 2020a). However, genetic studies do not support a causal effect of lipoprotein(a) levels on the risk of venous thromboembolism (Helgadottir et al., 2012; Kamstrup et al., 2012; Danik et al., 2013).

*Safety*. Despite efficacy, a main requirement of a novel therapy is safety. As remarkably low LDL-C levels are achievable in a large proportion of patients treated with PCSK9 inhibitors, the question of safety refers not only to the safety of the drug, but also to the safety of very low LDL-C levels. Lowering the levels of other cardiovascular risk factors such as blood pressure or glucose is beneficial, but lowering blood pressure or glucose below an optimal range leads to increasing rates of adverse events (J-curve effect). The question of safety of evolocumab and very low LDL-C levels was addressed by several analyses. One prespecified analysis compared adverse events stratified by achieved LDL-C levels. While there was a monotonic relationship of achieved LDL-C with the primary endpoint, no association between achieved LDL-C level and serious adverse events and prespecified safety events, including, among others, diabetes, cataract, malignancies, and hemorrhagic strokes, was observed (Giugliano et al., 2017c). The specific question of neurocognitive disorders was addressed in a sub-study of FOURIER called EBBINGHAUS. Using the *Cambridge Neuropsychological Test Automated Battery*, 1,204 patients were investigated at baseline, at week 24, yearly, and at the end of the study. In none of the defined endpoints, significant changes occurred in patients treated with evolocumab compared to patients treated with placebo. No association between cognitive changes and LDL-C level was observed (Giugliano et al., 2017b). Apart from this subset of patients, all patients in FOURIER were asked to complete a 23-item survey on memory and executive functions from the *Everyday Cognition* scale at the end of the study. 22,655 patients completed this form. Patients treated with evolocumab reported declines in cognitive function in a similar proportion (3.7%) as patients who had received placebo (3.6%). Furthermore, among the patients achieving LDL-C <20 mg/dL at week 4, a decline in cognitive function was not more often reported than by patients with achieved LDL-C ≥100 mg/dL (Gencer et al., 2020a). Overall, within the follow-up period of FOURIER, treatment with evolocumab and achieving very low LDL-C levels did not appear to be accompanied by any adverse medical events.

#### ODYSSEY Outcomes Study

The ODYSSEY Outcomes study (Schwartz et al., 2018) represents the third landmark trial of antibodies against PCSK9. There are some differences compared to the FOURIER trial such as the study population and the possibility of dose adjustments (Table 2).

##### Study population, treatment protocol, endpoints

Patients were eligible for inclusion in the ODYSSEY Outcomes trial if they had a history of an acute coronary syndrome within 1–12 months before randomization. High-intensity statin therapy was encouraged with doses of at least atorvastatin 40 mg or rosuvastatin 20 mg. Patients were randomized to alirocumab 75 mg every 2 weeks or placebo subcutaneously in a 1:1 ratio. In contrast to the FOURIER trial in which no dose adjustments were allowed, an LDL-C of 25–50 mg/dL was targeted in the ODYSSEY Outcomes trial, and LDL-C values below 15 mg/dL were to be avoided. Dose adjustments or even replacement of alirocumab with placebo were done under blinded conditions. The primary endpoint was a composite of coronary death, myocardial infarction, ischemic stroke, and unstable angina requiring hospitalization.

##### Results

In the ODYSSEY Outcomes trial, 18,924 patients with a mean age of 59 years (25% women) were included. In 83% of the patients, the acute coronary syndrome qualifying for inclusion was myocardial infarction. The median time from the qualifying event to inclusion was 2.6 months. Hypertension was present in ∼65% of the patients, diabetes mellitus in 29%, and 24% were current smokers. 89% of the participants received at least atorvastatin 40 mg or rosuvastatin 20 mg. The mean baseline LDL-C level was 92 mg/dL.

At 12 months of treatment, LDL-C was reduced under alirocumab to a mean of 48 mg/dL; at 48 months of treatment, mean LDL-C was 66 mg/dL. Non-HDL cholesterol and apolipoprotein B were similarly reduced.

After a median follow-up of 2.8 years, the relative risk for the primary endpoint was reduced by 15% with a hazard ratio of 0.85 (95% CI, 0.78–0.93), although death from CAD or from cardiovascular causes was not. In the contrary, all-cause mortality was significantly lower under alirocumab, however, this finding is considered nominally significant as to the hierarchical testing with previous non-significant endpoints. The same is true for the nominally significantly reductions in the risk of myocardial infarction, ischemic stroke, unstable angina, and coronary revascularization.

As with evolocumab, the frequency of adverse events was similar in the alirocumab and placebo groups with the exception of injection-site reactions, which were more common under alirocumab (3.8 vs. 2.1%) but were generally mild and led to the discontinuation of treatment in only in a few patients.

##### Secondary and post hoc analyses

As with the FOURIER study, several secondary and *post hoc* analyses with data from the ODYSSEY Outcomes study have been conducted.

*Subgroups*. In general, similar to the FOURIER study, patients with higher cardiovascular risk gained greater absolute risk reductions from treatment with alirocumab.

One factor qualifying for higher cardiovascular risk is previous coronary artery bypass grafting (CABG). While patients without previous CABG experienced an absolute risk reduction of 1.3% and those with CABG *after* the qualifying acute coronary syndrome event of 0.9%, patients with CABG *before* the qualifying acute coronary syndrome had an absolute risk reduction of 6.4%. The relative risk reductions were comparable and consistent with the overall results of the trial (Goodman et al., 2019).

Another marker of cardiovascular risk, polyvascular disease, showed a similar pattern. The event rate differed largely depending on the number of affected artery beds. Patients with isolated coronary atherosclerosis had an event rate of 10.0% during follow-up (placebo group), those with two affected artery beds (peripheral artery disease or cerebrovascular atherosclerosis additionally) had an event rate of 22.2%, and those with three affected artery beds of 39.7%. The corresponding absolute risk reductions were 1.4, 1.9, and 13.0%. The absolute reduction in risk of death was an impressive 16.2% in patients with atherosclerosis in three vascular beds (Jukema et al., 2019a).

Prevalent diabetes also considerably affected cardiovascular risk: In the placebo group, patients without diabetes at baseline had an event rate during follow-up of 8.5%, those with prediabetes of 9.2%, and those with diabetes of 16.4%. While the relative risk reductions were similar in the three groups, the absolute risk reductions were 1.2% for patients without diabetes or with prediabetes, and 2.3% in patients with diabetes. Similar to evolocumab, alirocumab did not affect the incidence of new-onset diabetes (Ray et al., 2019).

In another analysis, a polygenic risk score was used to identify patients with high genetically determined cardiovascular risk. These patients had greater absolute and relative risk reductions after treatment with alirocumab compared to those with lower scores (Damask et al., 2020).

Lastly, alirocumab reduced cardiovascular risk independently of age. Importantly, older patients did not experience more adverse events associated with alirocumab treatment, and the absolute risk reduction was larger (Sinnaeve et al., 2019).

*Lipoprotein(a)*. Alirocumab reduces lipoprotein(a) in a similar fashion as evolocumab. A pre-specified analysis found that both baseline lipoprotein(a) and LDL-C predicted the primary endpoint (Bittner et al., 2020). Furthermore, their respective reductions by alirocumab were independent predictors of major adverse cardiovascular events. It was therefore concluded that – although the absolute reductions in lipoprotein(a) were small, owing to the fact that baseline median lipoprotein(a) was only 21.2 mg/dL – lowering of lipoprotein(a) by alirocumab is an independent contributor to the observed reductions in cardiovascular risk under treatment. Furthermore, the effect of alirocumab on lipoprotein(a) was investigated with respect to venous thromboembolic and peripheral artery disease events. Higher lipoprotein(a) was associated with higher risk of peripheral artery disease, and the risk of respective events was lowered by alirocumab treatment, with greater risk reductions in patients with higher lipoprotein(a). In contrast to evolocumab, the reduction in risk of venous thromboembolism by alirocumab did not reach statistical significance (hazard ratio 0.67 [95% CI, 0.44–1.01]). The change in lipoprotein(a) by alirocumab was associated with the risk of venous thromboembolism (Schwartz et al., 2020). Again, however, these data only suggest but do not prove a possible causal association.

*Outcomes*. Typical outcome trials are designed to show a benefit in reducing the first cardiovascular event after inclusion in the trial, however, preventing subsequent cardiovascular events is clinically meaningful as well. Compared to 3,064 first events, there were a total of 5,425 events in the ODYSSEY Outcomes trial. Compared to placebo, 190 fewer first events, and 385 fewer total events occurred under alirocumab, indicating greater benefits from treatment as opposed to only taking into account the first events (Szarek et al., 2019).

The reduction in mortality was investigated in a subgroup of patients with follow-up of at least 3 years (*n* = 8242). In this group, all-cause mortality was significantly reduced (hazard ratio 0.78 [95% CI, 0.65–0.94]; *p* = 0.01). Patients with high baseline LDL-C and low achieved LDL-C benefitted the most. This analysis therefore suggests that PCSK9 inhibition has the potential to reduce mortality after acute coronary syndrome with longer treatment than in the main study, and especially in patients with elevated risk due to high LDL-C (Steg et al., 2019).

In another study, the authors investigated the specific type of myocardial infarction prevented by treatment with alirocumab (White et al., 2019). As expected, treatment reduced the risk of the “classic” myocardial infarction which is due to spontaneous plaque rupture. Additionally, and for the first time for any lipid-lowering therapy, it could be demonstrated that also the risk of type 2 infarction was reduced as well, a finding possibly explained by a reduction of progression of atherosclerosis, similar to what was found in the above-mentioned GLAGOV study (Nicholls et al., 2016).

Apart from myocardial infarction, alirocumab also reduced the risk of stroke. Specifically, the composite of any type of stroke was significantly reduced, as were ischemic strokes. Hemorrhagic strokes tended to occur less frequently under alirocumab; importantly, there was no suggestion of increased risk of cerebral hemorrhage under alirocumab and the achieved LDL-C levels. The reduction in risk of stroke was independent of prevalent cerebrovascular disease (Jukema et al., 2019b).

### Ongoing Studies

The efficacy of evolocumab in reducing cardiovascular risk is currently being investigated in the VESALIUS study in a cohort with high or very high cardiovascular risk, but without previous myocardial infarction or stroke (NCT03872401) (ClinicalTrials.gov, 2020c). Patients are eligible for inclusion if they have CAD with significant stenoses or previous percutaneous or operative revascularizations, significant carotid stenoses, significant peripheral artery disease, or diabetes. The presence of additional cardiovascular risk factors is required. The investigators plan to allocate 12,000 participants to either evolocumab or placebo. Follow-up will be at least 4 years.

## Novel Approaches to Inhibit PCSK9

The development and demonstration of clinical efficacy of PCSK9 antibodies within 15 years of the first description of PCSK9 and its involvement in lipid metabolism is exemplary. Based on the large outcome trials FOURIER and ODYSSEY Outcomes, current major guidelines recommend PCSK9 antibodies for patients not achieving their LDL-C target (Grundy et al., 2018; Mach et al., 2020).

However, there are also drawbacks in the use of antibodies such as evolocumab and Alirocumab. One main issue is the high price of these therapeutics. Cost-efficacy analyses of PCSK9 antibodies come to differing conclusions (Kazi et al., 2017, 2019; Lee et al., 2018; Brunetti et al., 2019; Dressel et al., 2019; Bhatt et al., 2020). An undisputed fact from an economic point of view is that prescription of PCSK9 antibodies will be most effective in patients with the highest cardiovascular risk.

Alternatively, approaches to inhibit PCSK9 other than the use of antibodies may be able to provide the same (or even more) advantages as antibodies at a lower production cost. Different approaches have been pursued, among which are small molecules, adnectins, and active vaccination (Table 3; Urban et al., 2013; Elbitar et al., 2016; Nishikido and Ray, 2018; Catapano et al., 2020). However, among all alternatives to antibodies, targeting of PCSK9 mRNA with the small interfering RNA inclisiran is at the most advanced stage of development. Key studies investigating the use of this agent are described below.

**TABLE 3:** Pharmacologic approaches to target PCSK9.

**Class**	**Mechanism(s) of action**	**State of development**
Monoclonal antibodies	Extracellular binding and neutralization of PCSK9	Positive outcome trials, evolocumab and alirocumab approved for clinical use
Small interfering RNA	Inhibition of intracellular PCSK9 production by PCSK9 mRNA degradation	Ongoing outcome trial with inclisiran, under approval
Antisense oligonucleotides		Terminated/preclinical
Vaccines	Induction of immune response against PCSK9	Phase 1
Adnectins	Binding of the LDL receptor-interacting PCSK9 region	Phase 1/2
Mimetic peptides	Competitive inhibitors mimicking the PCSK9-binding domain of the LDL receptor	Preclinical
Small molecules	Inhibition of PCSK9-LDL receptor interaction	Preclinical
	Inhibition of PCSK9 mRNA translation by blocking of the ribosome	
	Blocking the binding of PCSK9 to the LDL-LDL receptor complex	
	Blocking the interaction of PCSK9 and heparan sulfate proteoglycans which is necessary for the PCSK9-LDL receptor complex formation	

*Abbreviation: LDL, low-density lipoprotein. Data from Urban et al. (2013), Elbitar et al. (2016), Nishikido and Ray (2018), and Catapano et al. (2020).*

### LDL-C Lowering With Inclisiran

#### Physiology

Inclisiran (previously known as ALN-PCSsc) is a small interfering RNA. The molecule is a modified double-stranded RNA conjugated with triantennary *N*-acetylgalactosamine (GalNAc). Through the GalNAc moiety, after subcutaneous administration, inclisiran is specifically taken up by hepatocytes through binding of the asialoglycoprotein receptor, which is abundantly expressed on hepatocytes. After endosomal uptake, a small part of the drug is released into the cytoplasm. The RNA strands dissociate into a passenger strand and a guide strand. The guide strand, which has a complementary sequence to PCSK9 mRNA, recruits several proteins that assemble the RNA-induced silencing complex (RISC). This complex binds PCSK9 mRNA and leads to its degradation (Figure 2B1). Once bound in the RISC, one single guide strand can bind and degrade several PCSK9 mRNA strands over a prolonged period of time (Katzmann et al., 2020).

#### Clinical Data

After promising phase 1 trials, the first large phase 2 trial was the ORION-1 study which included 501 patients at high cardiovascular risk with LDL-C levels greater than 70 mg/dL. Statin therapy at the highest tolerable dose was encouraged. Inclisiran lowered PCSK9 and LDL-C levels in a dose-dependent manner. After 2 doses of inclisiran, LDL-C was reduced by up to 53% at day 180 (Ray et al., 2017).

In the clinical trials of inclisiran (phase 3), the agent was administered at a dose of 300 mg subcutaneously initially, after 3 months, and every 6 months thereafter. Three phase 3 trials of inclisiran have recently been published. In the ORION-9 study, 482 patients with HeFH were included (Raal et al., 2020). HeFH had to be diagnosed either genetically or clinically according to the Simon Broome criteria. LDL-C level had to be at least 100 mg/dL on the maximally tolerable statin dose with or without ezetimibe. Treatment with PCSK9 antibodies was not allowed. Patients were randomized to inclisiran 300 mg or placebo in an 1:1 fashion. Inclisiran reduced LDL-C from a baseline of 153 mg/dL by 47.9% at day 510 after randomization. The effects were independent of the underlying genotype. Adverse and serious adverse events occurred in similar frequencies in the inclisiran and placebo groups.

The ORION-10 and ORION-11 trials were similar in design (Ray et al., 2020). While ORION-10 was conducted in the United States and included only patients with established ASCVD, ORION-11 was conducted in Europe and South Africa and included patients with ASCVD or ASCVD risk equivalents. LDL-C levels had to be at least 70 mg/dL (100 mg/dL in patients with ASCVD risk equivalent) on maximally tolerated lipid-lowering therapy except for PCSK9 antibodies. Together, a total of 3,178 patients were included in both studies. Randomization was 1:1 to inclisiran or placebo. Baseline LDL-C level was 105 mg/dL (ORION-10) and 106 mg/dL (ORION-11). Inclisiran reduced LDL-C level by 52.3 and 49.9% at day 510. Injection-site reactions were more common under inclisiran (2.6 vs. 0.9% in ORION-10, 4.7 vs. 0.5% in ORION-11), but were generally mild and not severe or persistent in any case.

The ORION-9, -10, and -11 trials share some similarities. In all studies, the majority (∼90%) of patients were on statin therapy. Inclisiran not only lowered LDL-C, but also total and non-HDL cholesterol, apolipoprotein B, triglycerides, and lipoprotein(a), and increased HDL cholesterol. Adverse events were generally mild, with injection-site reactions being the most frequently side effect.

Currently, the cardiovascular outcome study ORION-4 is ongoing. 15,000 patients with established ASCVD, defined as previous myocardial infarction, stroke, or peripheral artery disease with previous intervention, are randomized to inclisiran or placebo. The trial duration is five years, completion is expected in 2024 (NCT03705234) (ClinicalTrials.gov, 2020a).

The available studies to date suggest that inclisiran is a well-tolerated and effective LDL-C-lowering agent with the major advantage of infrequent administration of twice yearly.

### Comparison of Inclisiran and Antibodies Against PCSK9

If the ongoing cardiovascular outcome trial will demonstrate superiority of inclisiran compared to placebo with respect to cardiovascular outcomes and assuming a good long-term safety profile, inclisiran will be an important therapeutic alternative to PCSK9 antibodies. While the latter have to be administered every 2 weeks, inclisiran is administered twice yearly only, a fact that could potentially be of advantage in terms of medication adherence. The process of development of small interfering RNAs is potentially less complicated than that of antibodies, and therefore, the production and subsequently the drug costs may be lower (Katzmann et al., 2020).

On the other hand, one should note that the two aforementioned strategies of targeting PCSK9 are fundamentally different: While PCSK9 antibodies bind only extracellular PCSK9 and do not interfere with its intracellular form (Figure 2B2), inclisiran blocks the synthesis of PCSK9, albeit mainly in the liver. Since intracellular functions of PCSK9 have been reported, such as inducing LDL receptor degradation by affecting its intracellular trafficking distinct of the extracellular pathway (Poirier et al., 2009; Schulz et al., 2015; Glerup et al., 2017; Schlüter et al., 2017). As with any new therapeutic principle, the long-term safety of the drug as well as the presence of potential off-target effects need to be closely monitored. While there is currently no indication of a clinical relevance of these concerns, long-term data are wanted.

## Concluding Remarks

Inhibition of PCSK9 with monoclonal antibodies has emerged as the third cornerstone in addition to statins and ezetimibe in treating elevated LDL-C levels and preventing ASCVD. Major international guidelines recommend the use of PCSK9 antibodies in patients at elevated cardiovascular risk not achieving their respective LDL-C targets (Grundy et al., 2018; Mach et al., 2020). Evolocumab and alirocumab reduce LDL-C by 60% in a broad range of populations either as monotherapy or in addition to statins and/or ezetimibe. The reported adverse event profile is excellent except for slightly more injection-site reactions than placebo. Patients at the highest cardiovascular risk gain the greatest absolute risk reductions by treatment with antibodies against PCSK9, guiding patient selection.

The FOURIER and ODYSSEY Outcomes studies had important implications for guideline recommendations. Specifically, based on the fact that patients achieving lower LDL-C levels than previously recommended gained further cardiovascular benefits, the ESC/EAS guidelines published in 2019 now recommend lower treatment targets for LDL-C depending on cardiovascular risk, for example 55 mg/dL in patients at very high cardiovascular risk such as patients with CAD (Mach et al., 2020). Taken together, the study results suggest that for LDL-C levels, it does not appear that a value exists that is too low regarding safety und which therefore, should be avoided (Katzmann and Laufs, 2019). Studies with even longer follow-up such as the Fourier OLE trial (NCT03080935) (ClinicalTrials.gov, 2020d) are currently underway to further verify and establish this observation.

Novel approaches to inhibit PCSK9 are under clinical assessment. The most advanced in development is the small interfering RNA inclisiran, which is currently being tested in a large cardiovascular outcome trial (NCT03705234). siRNA approaches – assuming both positive study results and an acceptable safety profile – may provide a valuable addition to the growing armamentarium for an individualized treatment of dyslipidemias (Katzmann et al., 2020).

## Author Contributions

JK wrote the initial draft of the manuscript. IG-B and UL contributed important intellectual content and revised the article. All authors have read and approved the final version of the manuscript.

## Conflict of Interest

IG-B reports personal fees and non-financial support from Akcea, Amgen, and Sanofi, and personal fees from Aegerion, Daiichi Sankyo, and Regeneron, outside the submitted work. UL has received fees from Amgen, Daiichi Sankyo, Novartis, and Sanofi outside the submitted work. The remaining author declares that the research was conducted in the absence of any commercial or financial relationships that could be construed as a potential conflict of interest.
